# ASPHER Statement: 5 + 5 + 5 Points for Improving Food Security in the Context of the Russia-Ukraine War. An Opportunity Arising From the Disaster?

**DOI:** 10.3389/phrs.2022.1605321

**Published:** 2022-09-21

**Authors:** Henrique Lopes, Jose Martin-Moreno

**Affiliations:** ^1^ Public Health Unit, Health Sciences Institute, Catholic University of Portugal, Lisbon, Portugal; ^2^ Department of Preventive Medicine and Public Health, Medical School and INCLIVA, University of Valencia, Valencia, Spain; ^3^ ASPHER Honours’ Committee, Brussels, Belgium

**Keywords:** food security, Russia-Ukraine war, ASPHER, public health, education, public policies

## Statement of the Association of Schools of Public Health in the European Region (ASPHER)

The situation triggered by the war initiated by Russia in Ukraine, in addition to the widely known impacts that it has directly on the affected populations, has consequences that affect to almost the entire world population. The effects are verified either through increases in the reference prices of commodities, or by occasional ruptures in the supply chains, all of which contribute to the worsening of the economic and social conditions of most of the people.

One of the sectors with predictably the greatest impact is food, through multiple mechanisms that end up having the same end result: the worsening of the quantity of food produced on the planet and potentially the decrease of diets quality.

Russia and Ukraine are two of the biggest food exporters at a worldwide level, as illustrated by the WPF [1], accounting for 14% of world exports of maize, 18% of wheat and 40% of sunflower. The immediate consequence of the war was the rise in the prices of essential products, both for human and animal food, with historic high values being registered. There have also been financial speculative phenomena with food commodities, which amplifies the phenomenon of price elasticities [2–5]. Several countries depend on a large scale on imports from these countries at war, especially those in African and in the Near East [6, 7].

Therefore, there are strong reasons to believe that in addition to potential food shortages in the war zone, there may be a worsening of the food condition in the near future in regions where it was already fragile or perilous. Even for countries with sufficient economic conditions to continue to obtain supplies at rising prices of food raw materials, it must be considered that many vulnerable groups already faced difficulties before the war in maintaining a sufficient, healthy and balanced diet.

In addition to the above-mentioned situation, Russia is the world’s largest exporter of fertilizers [8–12], particularly in the supply chain to Western Europe, a condition that has now been interrupted by countersanctions [13, 14] to the country. Given that it is not a type of product that can be easily found elsewhere and as it is directly indexed to energy prices, which are also rising, it is expected that in this agricultural year producers will find less availability of fertilizers on the market and those that are available are sold at very high prices. This also leads in the long term to a decrease in agricultural production outside the conflict zone.

The months of March and April are critical for the volume and quality of European crops and plantations when many sowings are fertilized. The failure at this point ends up having repercussions later, since not sowing or not fertilizing now will prevent or reduce the expected harvests in the coming months of July–September. This means that even assuming a rapid resolution of the conflict, problems in the world food supply chain are expected until August 2023.

The lack of food in the global supply chain always ends up triggering an increase in demand for new agricultural land, reinforcing deforestation, the use of less suitable soils for agriculture, an increase in the consumption of wild species, overfishing, and other protein- and carbohydrate-seeking behaviors.

This global picture of the future of human and animal nutrition poses complex challenges to Public Health, but also opens the door to developments that would have been unthinkable until recently.

We should now promote food education programs among populations around the world to reduce the amount of food commodities needed to provide the same final amount of food, reduce costs per final kilogram and improve the diet provided to each person, thereby promoting better health. Therefore, the Association of Schools of Public Health in the European Region (ASPHER) calls for reinforcement of two main pillars of action: Education and Public Policies.

## Education Actions

(1) Taking advantage of the food crisis to move towards healthier diets and thus combat the metabolic diseases that are highly prevalent in the wealthiest countries.
(2) Combating food waste, with education interventions focused on reducing food losses along the supply chain (currently in immense amounts).
(3) Regaining appreciation for local agricultural production and products with less commercial marketing, but with great food value. History shows that already happened in previous conflicts.
(4) Considering options that integrate what habitually were considered wasted parts of the animals and plants, thus reducing the pressure in the search for other food sources.
(5) Fostering the search for alternatives to animal proteins that require a high consumption of cereals per kilogram of final production by others of lesser consumption.

## Public Policies Actions

(1) Balancing the production of biofuels using missing food commodities with their allocation for food purposes.
(2) Suppressing bans on of food based on its “beauty,” such as the “ugly fruit,” non-standard size vegetables and other similar measures aimed at reducing food waste.
(3) Protecting wild areas from the rampant expansion of agricultural land, including protecting forests and controlling overfishing to temporarily compensate for the lack of food. There are many technological resources that can be used in agriculture and livestock capable of optimizing the production, transport, and use of food since the immediate search for profit is not the main reason for production, but the search for the best possible food for humans.
(4) Valuing foods that are currently undervalued in the world food supply chain for commercial reasons, but which have a high nutritional value.
(5) Adding to the current diet resources that are currently only marginal in human and animal nutrition, such as edible algae, herbs still considered harmful in most countries (e.g., purslane and nettles, which have very high food value).

These measures aim to:

(1) Prevent the aggravation or appearance of famines resulting from the war between Russia and Ukraine outside the conflict area themselves potentially causing or potentiating conflicts in other planetary regions.
(2) Mitigate food shortages in the conflict zone and world-wide.
(3) Enhance the quality of the diet available to each citizen.
(4) Improve food education of each citizen.
(5) Reduce the impacts on the planet and therefore of climate change resulting from the production of human food.

## Author’s Note

The Association of Schools of Public Health in the European Region (ASPHER) is the key independent European organisation dedicated to improving and protecting public health by strengthening education and training of public health professionals for practice and research. www.aspher.org.

## Editorial Note

ASPHER is responsible and liable for the content of this statement. The statement has been reviewed for clarity by a PHR Editor-in-Chief but has not been externally peer reviewed.
